# Feasibility of biventricular volume and function assessment using first-pass gated ^15^O-water PET

**DOI:** 10.1186/s13550-018-0445-x

**Published:** 2018-09-17

**Authors:** Fayçal Ben Bouallègue, Denis Mariano-Goulart, Denis Agostini, Alain Manrique

**Affiliations:** 1 Nuclear Medicine Department, Montpellier University Hospital, Montpellier, France; 2 PhyMedExp, INSERM – CNRS, Montpellier University, Montpellier, France; 3 Nuclear Medicine Department, CHU de Caen, Caen, France; 4 UNICAEN, EA 4650 SEILIRM, GIP Cyceron, Normandie University, Caen, France

**Keywords:** ^15^O-water PET, First-pass, Ventricular volume, Ventricular function

## Abstract

**Background:**

We investigated the feasibility of left ventricular (LV) and right ventricular (RV) volume and function estimation using a first-pass gated ^15^O-water PET. This prospective study included 19 patients addressed for myocardial perfusion reserve assessment using ^15^O-water PET. PET data were acquired at rest and after regadenoson stress, and gated first-pass images were reconstructed over the time range corresponding to tracer first-pass through the cardiac cavities and post-processed using TomPool software; LV and RV were segmented using a semi-automated 4D immersion algorithm. LV volumes were computed using a count-based model and a fixed threshold at 30% of the maximal activity. RV volumes were computed using a geometrical model and an adjustable threshold that was set so as to fit LV and RV stroke volumes. Ejection curves were fitted using a deformable reference curve model. LV results were compared to those obtained using ^99m^Tc-sestamibi gated myocardial SPECT in terms of end-diastolic volume (EDV), end-systolic volume (ESV), stroke volume (SV), and ejection fraction (EF).

**Results:**

There was an excellent concordance between rest and stress PET in terms of EDV and ESV (Lin’s coefficient ~ 0.85–0.90), SV (~ 0.80), and EF (~ 0.75) for both ventricles. Correlation with myocardial SPECT was high for LV EDV (Pearson’s *R* = 0.89, *p* < 0.001) and ESV (*R* = 0.87, *p* < 0.001) and satisfying for LV SV (*R* = 0.67, *p* < 0.001) and EF (*R* = 0.67, *p* < 0.001). Minimal LV ESV overestimation (+ 4 mL, *p* = 0.03) and EF underestimation (− 4%, *p* = 0.01) were observed using PET.

**Conclusions:**

Biventricular volume and function assessment are achievable using the first-pass PET, and LV parameters correlate well with those derived from gated myocardial SPECT.

## Background

Left ventricle (LV) function assessment is of utmost importance in the initial evaluation and during follow-up of patients with LV systolic [1] or diastolic [2] dysfunction or receiving cardiotoxic chemotherapy [3]. Imaging right ventricle (RV) function, albeit technically more challenging and less routinely implemented, may provide relevant diagnostic or prognostic information in patients with heart failure [4], after inferior myocardial infarction [5], or in more specific disorders such as amyloidosis [6] or arrhythmogenic dysplasia [7]. Standard LV or RV function assessment requires the estimation of end-diastole (ED) and end-systole (ES) volumes as well as ejection fraction (EF), which can be achieved using various non-invasive imaging techniques including echocardiography, cardiac magnetic resonance (CMR), and isotopic techniques. In the frame of nuclear medicine, cardiac function is usually studied by means of gated equilibrium radionuclide angiography, using either planar techniques or single photon emission computerized tomography (SPECT) and ^99m^Tc-labelled red blood cells (RBC) [8–10]. LV function analysis using ^15^O-CO blood-pool positron emission tomography (PET) has been proven to be achievable [11]. LV functional parameters may alternatively be inferred from perfusion images obtained using dedicated tracers such as ^99m^Tc-sestamibi (for SPECT) [12], ^82^Rb [13] or ^13^N-ammonia [14] (for PET), or viability images obtained using ^18^FDG PET [15]. Most of these isotopic techniques have been thoroughly validated against CMR as the reference standard [8, 9, 11, 14–16].

Planar first-pass radionuclide angiography using ^99m^Tc-labelled agents has been extensively employed for decades for LV and RV function analysis [17]. The excellent count sensitivity of PET technology makes it an ideal means for dynamic tomographic imaging of fast transient phenomena such as radiotracer transit through cardiac cavities. The steadily increasing use of PET (and particularly cardiac PET) in clinical routine reinforces the motivation to develop first-pass PET methods for cardiac function evaluation. The feasibility of cardiac output and stroke volume (SV) estimations at first-pass by means of the Stewart-Hamilton indicator-dilution principle has already been demonstrated using either ^82^Rb [18], ^18^FDG [19], or ^15^O-water PET [20], the provided estimates being in close agreement with those of RBC-SPECT, echocardiography, and CMR. Todica et al. described LV volume and function measurements in healthy rats using first-pass gated ^18^FDG PET [21], without significant difference compared to CMR in terms of ejection fraction. Such cardiac function assessment using first-pass gated PET has been poorly investigated in humans since only two studies are reported on the feasibility of LV function assessment using first-pass gated ^15^O-water PET [22, 23].

TomPool is a free software developed in Montpellier University Hospital and designed for semi-automated post-processing of equilibrium blood-pool SPECT images. It has previously been validated against CMR for LV and RV function assessment [8]. It relies on a watershed immersion algorithm for LV and RV segmentation and a deformable reference model for time-activity curve fitting [24, 25].

The aim of the present work was to investigate the feasibility of LV and RV volume and function measurement using the first-pass^15^O-water PET in a cohort of patients referred for myocardial perfusion assessment at rest and after vasodilator stress. First-pass PET was validated against gated myocardial SPECT for LV measurements.

## Methods

### Patients

Nineteen patients aged 47–75 years (median 66 years, 14 men and 5 women) were prospectively recruited from the outpatients of the Nuclear Medicine Department at Caen University Hospital from December 2015 to November 2016 in the frame of the WATERDAY study (ClinicalTrials.gov unique identifier NCT02278497) aiming to compare myocardial perfusion and flow reserve using dynamic myocardial SPECT to ^15^O-water PET and endovascular fractional flow reserve measurement [26]. Inclusion criteria were the presence of at least one significant (≥ 50%), non-occlusive, coronary stenosis on percutaneous coronary angiography, and no history of myocardial infarction. All patients underwent both myocardial SPECT and ^15^O-water PET within 3–17 days (median 10 days), without any relevant cardiovascular event or change in cardiotropic medication between the two examinations. The study was approved by the Regional Ethics Committee (CPP Nord-Ouest III, France), and all patients gave written informed consent. The clinical characteristics of the study population are summarized in Table 1.

**Table 1:** Characteristics of the study population

Male gender	14 (74%)
Age (years)	65 ± 8 [47–75]
CCS angor class 1	13 (68%)
CCS angor class 2	6 (32%)
Cardiovascular risk factors	
BMI > 30 kg/m^2^	3 (16%)
Diabetes mellitus	5 (26%)
Hypertension	11 (58%)
> 3 cardiovascular risk factors	5 (26%)
Medical therapy	
Antiplatelet	17 (89%)
Beta-blocker	8 (42%)
ACE inhibitor or AT-II antagonist	10 (53%)
Calcium channel blocker	3 (16%)
Long acting nitrates	2 (11%)

Cardiovascular risk factors include obesity, diabetes mellitus, hypertension, dyslipidaemia, smoking, and family history

*CCS* Canadian Cardiovascular Society, *BMI* body mass index

### Myocardial SPECT acquisition and analysis

All SPECT acquisitions were carried out using a dedicated CZT cardiac SPECT camera (D-SPECT; Spectrum-Dynamics, Biosensors, Caesarea, Israel) with the patient in supine position. Rest and stress acquisitions were performed in the same session. Rest images were acquired during 5 min, 6-7 min after injection of 2.5 MBq/kg of ^99m^Tc-sestamibi. For stress imaging, 7 MBq/kg of ^99m^Tc-sestamibi was injected at peak hyperaemia following IV injection of 400 μg of regadenoson, and data were acquired 6–7 min later over 2 min. Gated SPECT images were reconstructed using the manufacturer’s dedicated software and post-processed using commercially available software (Corridor4DM, INVIA, Ann Arbor, MI) [27] to obtain LV end-diastolic volume, end-systolic volume, stroke volume, and ejection fraction. The *R*-*R* interval was divided into 16 intervals. Myocardial wall segmentation was fully automated, and additional slight manual adjustment of LV basal limit was performed whenever necessary.

### ^15^O-water PET acquisition and analysis

All PET acquisitions were achieved using a GE Discovery VCT RX scanner (GE Healthcare, Buc, France) and started with a low-dose transmission CT scan for attenuation correction. Two intravenous injections of ^15^O-water (1.5 to 3 MBq/kg) were performed, each one being simultaneously performed with the start of a dynamic gated 4′30″ emission scan. The second emission scan was performed at peak vasodilator stress following IV injection of 400 μg regadenoson. After correction for random coincidences, scatter, and dead time, the acquired list-mode data was reconstructed twice. First, a dynamic 24-frame sequence (14 × 5″, 3 × 10″, 3 × 20″, and 4 × 30″) was obtained using Fourier rebinning and 2D filtered back-projection. It allowed to extract a time-activity curve (TAC) in a region of interest (ROI) centred on the cardiac area. TACs were characterized by a sharp early peak corresponding to tracer first-pass through the right then left cardiac cavities followed by a sustained decreasing plateau corresponding to myocardial uptake then wash out. End of tracer first-pass was detected by visual inspection as the TAC transition point between the peak and the plateau. A second image reconstruction was then performed over the first-pass time range using 3D OSEM (nine subsets, two iterations) with eight-interval gating. Reconstructed images were re-oriented into cardiac canonical axes using CardIQ software on a dedicated workstation (Advantage Windows 4.4, GE Healthcare, Buc, France). Final image matrices were sampled on a 64 × 64 × 47 grid with 3.27 mm cubic voxels.

First-pass gated blood-pool images were post-processed using in-house software (TomPool, freely available for download at http://scinti.edu.umontpellier.fr) that was originally designed for equilibrium radionuclide angiography and adapted for first-pass data processing. Initial operator intervention was required to set the position of the septal plane (on horizontal long axis images), the valve plane (on vertical long axis images), and the infundibular plane as the upper limit of the RV (on short-axis images). A four-dimensional (4D) immersion algorithm was then run that produced a partition of the gated images into 4D regions centred on local intensity maxima. Each region was then assigned either to LV, RV, or extra-ventricular activity depending on the relative position of its barycentre with respect to the reference planes. Manual correction for misassigned regions was possible as well as semi-automated segmentation refinement. LV segmentation mask was obtained using a thresholding method at a fixed percentage of the 4D maximal intensity value inside the regions belonging to LV. Thresholds ranging from 20 to 40% were tested in order to determine the optimal threshold value. LV volumes were computed using a count-based method in which the volume of each voxel was normalized by the ratio of its intensity to the LV maximal intensity. RV segmentation mask was obtained by applying an adjustable threshold with respect to the 4D maximal intensity value inside the regions belonging to RV, and RV volumes were computed using a geometric method in which each voxel accounted for a constant volume regardless of its intensity. The RV threshold was chosen by the operator based on visual inspection and so as to fit as much as possible RV and LV stroke volumes (which was legitimate since no patient had documented valvular disease). Ventricular ejection curves were fitted to the eight time samples ({*t*_*n*_, *v*_*n*_}, *n* = 1…8) using a deformable curve model *v*_*n*_ = *η*[*R*(*t*_*n*_ + *τ*(*t*_*n*_))] where *R*(*t*) is the reference curve derived from a series of normal patients sampled over 512 time points, *η* a second-order polynomial, and *τ* a weighted sum of cosine functions with variable phase shift and frequency.

### Statistical analysis

Continuous variables are expressed as mean ± standard deviation (min-max range). Concordance and correlation between rest and stress functional parameters obtained using first-pass PET were assessed using Pearson’s correlation coefficient (*R*), Lin’s concordance correlation coefficient (ccc), and Bland-Altman analysis. Comparison between LV volume and function given by first-pass PET and those given by myocardial SPECT was achieved using the same metrics. Difference between homologous variables was characterized using a paired Student’s *t* test after checking data sample normalcy using the Kolmogorov-Smirnov test. All statistical computations were carried out using Excel (Microsoft, Redmond, WA).

## Results

Injected activities were 399 ± 88 MBq (175–521) for rest PET, 300 ± 81 MBq (131–459) for stress PET, 295 ± 57 MBq (169–419) for rest SPECT, and 774 ± 128 MBq (558–1012) for stress SPECT. Subject haemodynamic parameters are detailed in Table 2. All patients were in sinus rhythm, and no significant cardiac arrhythmia was observed in any SPECT or PET study (*R*-*R* interval rejection = 0% for all PET acquisitions and < 10% for all SPECT acquisitions).

**Table 2:** Subject haemodynamic parameters in PET and SPECT studies

	PET		SPECT	
	Rest	Stress	Rest	Stress
Heart rate (bpm)	69 ± 13	82 ± 24*	68 ± 15	88 ± 21*
Systolic BP (mmHg)	116 ± 15	118 ± 20	129 ± 21^ǂ^	134 ± 21^ǂ^
Diastolic BP (mmHg)	60 ± 10	59 ± 14	68 ± 12^ǂ^	73 ± 12^ǂ^
Rate-pressure product	8000 ± 1540	9810 ± 3890*	8770 ± 2380	11,780 ± 3500 *^ǂ^

*BP* blood pressure

*Significantly different from rest value (*p* < 0.05)

^ǂ^Significantly different from PET value (*p* < 0.05)

Visual analysis of perfusion myocardial SPECT data did not reveal advanced ischaemia (i.e., affecting more than two myocardial segments) in any of the 19 patients. An antero-septo-apical scar was evidenced in one patient.

Regarding first-pass PET, tracer bolus transition time through the cardiac cavities assessed on centred TACs was 25 ± 4 s (18–35) for rest acquisitions, corresponding to a total of 37 ± 12 (8–58) 10^6^ prompt events, and 22 ± 5 s (13–32) for stress acquisitions, corresponding to a total of 21 ± 8 (5–33) 10^6^ prompt events.

Table 3 details the results of first-pass PET data post-processing using TomPool (LV threshold at 30%) in terms of left and right ventricular end-diastolic volume, end-systolic volume, stroke volume, and ejection fraction. Mean value and range are indicated at rest and pharmacological stress for each parameter. Difference between stress and rest values (mean and its 95% confidence interval) is given in the third column. No statistically significant bias was observed between rest and stress volumes. SV and EF were minimally higher at stress for both LV (SV + 4.5 mL, *p* = 0.04; EF + 3%, *p* = 0.03) and RV (SV + 5.1 mL, *p* < 0.01; EF + 4%, *p* < 0.01). Stroke volumes for LV and RV were respectively 61 ± 13 mL versus 60 ± 12 mL at rest (not significant) and 65 ± 16 mL versus 65 ± 15 mL at stress (not significant).

**Table 3:** First-pass PET estimates of left and right ventricular volume and function at rest and after pharmacological stress. LV was segmented using a 30% threshold. The bias (difference between stress and rest value) is given along with its 95% confidence interval

	Rest PET		Stress PET		Difference	
	Mean ± SD	Range	Mean ± SD	Range	(Stress-rest)	
LV EDV (mL)	96 ± 28	60–161	98 ± 30	57–167	+ 1.9	[− 3.3, 7.2]
LV ESV (mL)	36 ± 18	12–77	33 ± 18	9–66	− 2.5	[− 6.3, 1.2]
LV SV (mL)	61 ± 13	39–84	65 ± 16	47–106	+ 4.5	[0.1, 8.8]*
LV EF (%)	65 ± 10	48–83	68 ± 10	49–89	+ 3.4	[0.4, 6.3]*
RV EDV (mL)	113 ± 28	70–175	115 ± 28	70–180	+ 2.1	[− 2.2, 6.3]
RV ESV (mL)	53 ± 19	24–82	50 ± 19	22–82	− 3.0	[− 6.3, 0.3]
RV SV (mL)	60 ± 12	43–93	65 ± 15	46–108	+ 5.1	[1.9, 8.2]**
RV EF (%)	54 ± 8	41–74	58 ± 9	42–77	+ 3.6	[1.7, 5.6]**

*LV* left ventricle, *RV* right ventricle, *EDV* end-diastolic volume, *ESV* end-systolic volume, *SV* stroke volume, *EF* ejection fraction

*p < 0.05; **p < 0.01

**Fig. 1:**
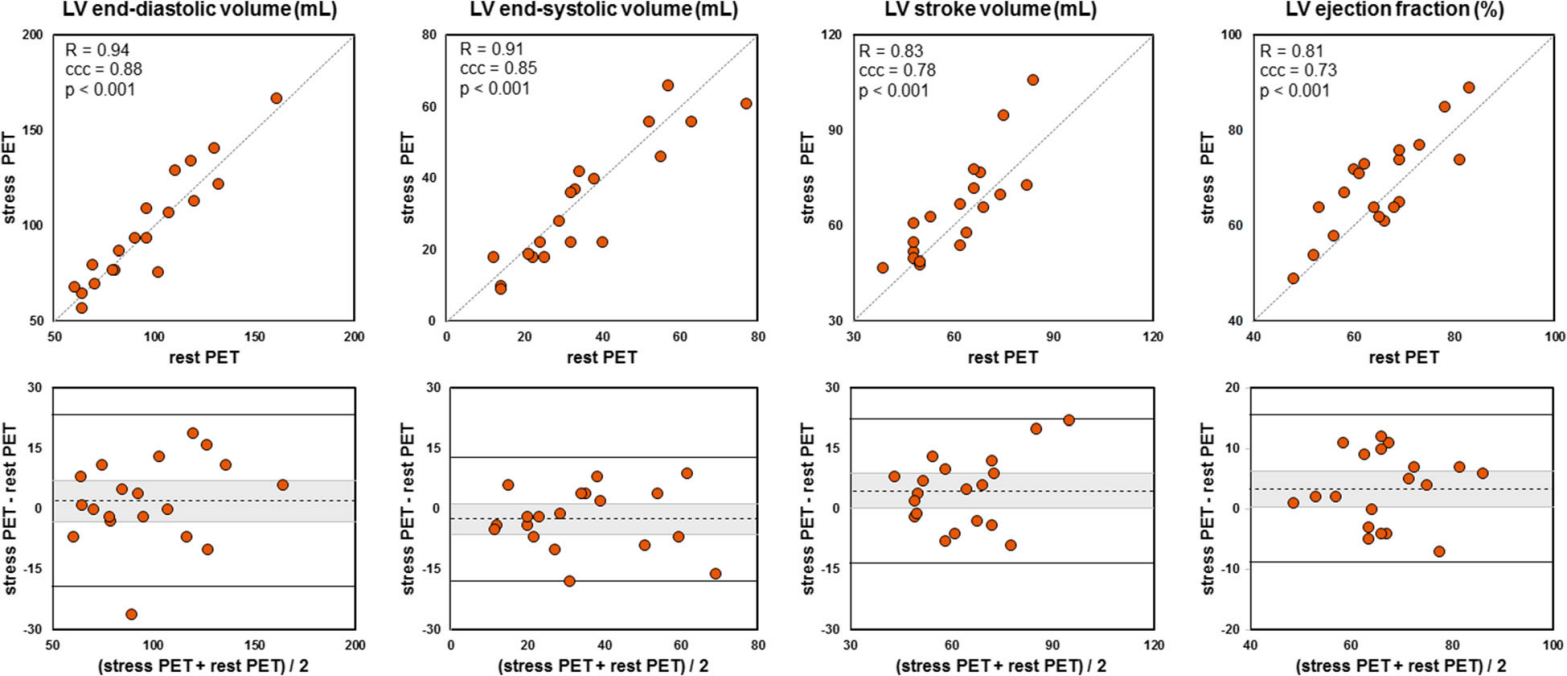
Comparison between rest and stress left ventricular volumes and function. Top: scatter plots. The grey lines stand for the perfect identity (*R*, Pearson’s correlation; ccc, Lin’s concordance correlation coefficient). Bottom: Bland-Altman diagrams. The dashed lines indicate the mean difference (greyed is the 95% confidence interval) and the plain lines the 95% limits of agreement

**Fig. 2:**
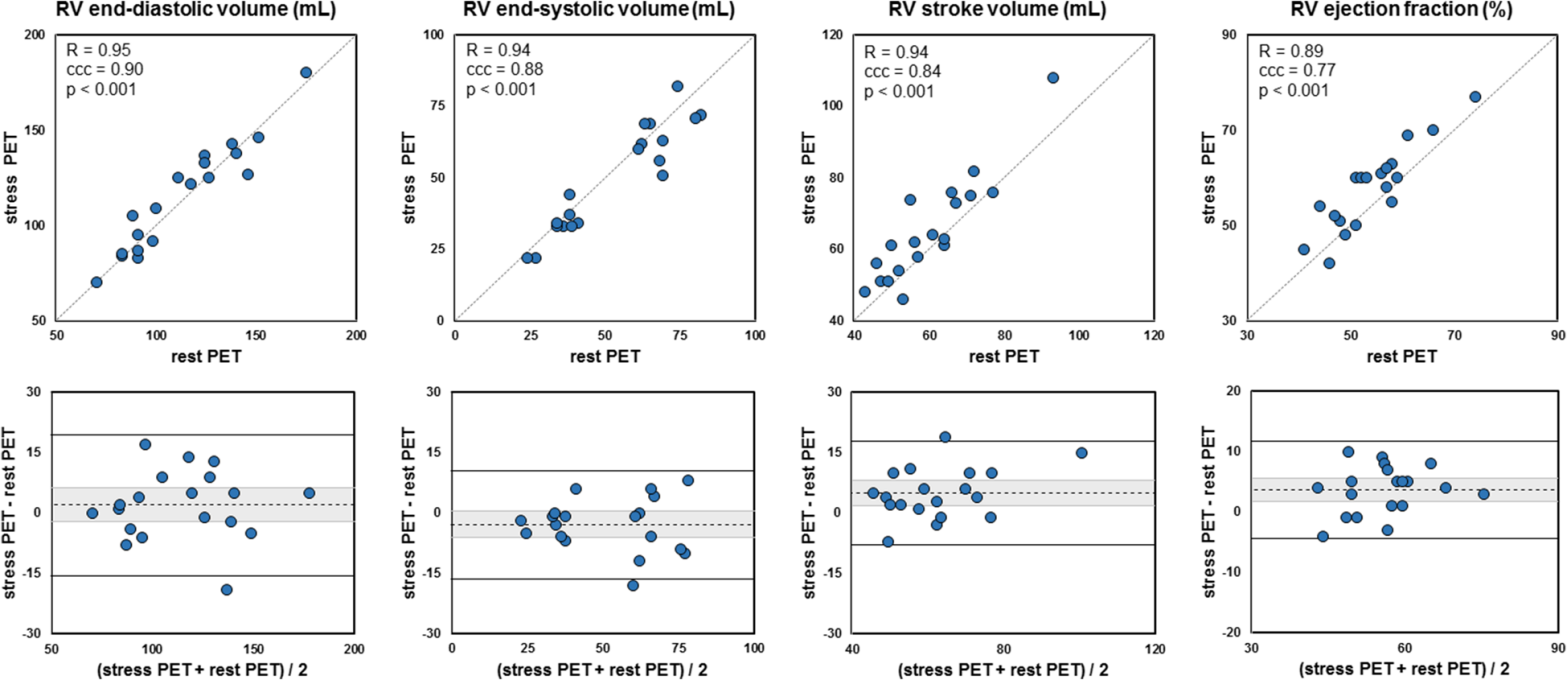
Comparison between rest and stress right ventricular volumes and function. Top: scatter plots. The grey lines stand for the perfect identity (*R*, Pearson’s correlation; ccc, Lin’s concordance correlation coefficient). Bottom: Bland-Altman diagrams. The dashed lines indicate the mean difference (greyed is the 95% confidence interval) and the plain lines the 95% limits of agreement

Figures 1 and 2 show the correlation and Bland-Altman plots comparing the volume and function measurements (from left to right: EDV, ESV, SV, and EF) between rest and stress studies for LV and RV, respectively. For both LV and RV, there was an excellent concordance between rest and stress results in terms of ED and ES volume (ccc ≥ 0.85, all *p* values < 0.001) and a substantial concordance in terms of ejection fraction (ccc = 0.73, *p* < 0.001 for LV; ccc = 0.77, *p* < 0.001 for RV).

Table 4 details the bias, correlation (Pearson’s coefficient), and concordance (Lin’s coefficient) between first-pass PET and myocardial SPECT LV functional parameters depending on the LV segmentation threshold (20 to 40% by steps of 5%). Overall, the 30% threshold appeared to achieve the best concordance with myocardial SPECT, except in terms of EF for which the 35% threshold performed slightly better.

**Table 4:** Bias, Prearson’s correlation (*R*), and Lin’s concordance (ccc) between first-pass PET and myocardial SPECT LV functional parameters according to the LV segmentation threshold

Threshold (%)	LV EDV (mL)			LV ESV (mL)			LV SV (mL)			LV EF (%)		
	Bias	*R*	ccc	Bias	*R*	ccc	Bias	*R*	Conc.	Bias	*R*	ccc
20	+ 12	0.89	0.83	+ 14	0.86	0.69	− 2	0.67	0.64	− 10	0.66	0.45
25	+ 7	0.89	0.86	+ 9	0.86	0.79	− 2	0.67	0.65	− 7	0.69	0.56
30	+ 1	0.89	0.88	+ 4	0.87	0.85	− 3	0.67	0.65	− 4	0.67	0.61
35	− 5	0.89	0.87	− 1	0.86	0.85	− 4	0.68	0.65	− 2	0.66	0.64
40	− 11	0.89	0.81	− 5	0.86	0.79	− 6	0.67	0.61	+ 1	0.65	0.63

*LV* left ventricle, *EDV* end-diastolic volume, *ESV* end-systolic volume, *SV* stroke volume, *EF* ejection fraction

**Fig. 3:**
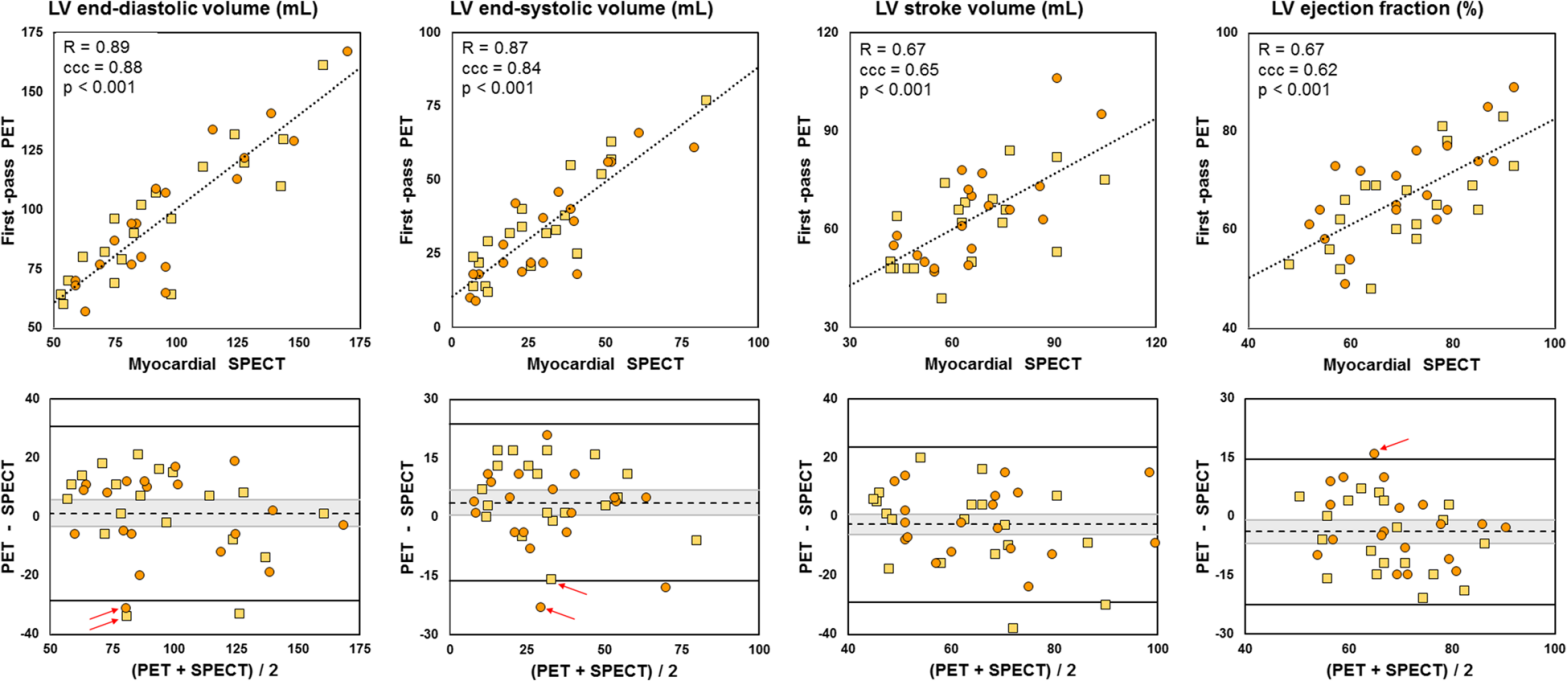
Correlation and agreement between left ventricular volume and function obtained using myocardial SPECT and first-pass PET. Yellow square markers stand for rest studies and orange round markers for stress studies. Top: scatter plots. The dotted lines stand for the linear regression (*R*, Pearson’s correlation; ccc, Lin’s concordance correlation coefficient). Bottom: Bland-Altman diagrams. The dashed lines indicate the mean difference (greyed is the 95% confidence interval) and the plain lines the 95% limits of agreement. The outliers labelled using red arrows refer to a single patient with an antero-septo-apical scar from prior infarct

Figure 3 displays both correlation and agreement between first-pass PET (LV threshold at 30%) and gated myocardial SPECT for LV volume and ejection fraction estimates. The scatter plot and the Bland-Altman diagram for each parameter (EDV, ESV, SV, and EF) are shown. The correlation was good for EDV and ESV estimations (respectively *R* = 0.89 and 0.87, *p* < 0.001) and fair for SV and EF estimations (*R* = 0.67, *p* < 0.001 for both). Compared to myocardial SPECT, first-pass PET tended to slightly overestimate EDV (+ 1 mL, not significant, limits of agreement [− 28, 31 mL]) and ESV (+ 4 mL, *p* = 0.03, limits of agreement [− 16, 24 mL]), and to moderately underestimate SV (− 3 mL, not significant, limits of agreement [− 29, 24 mL]) and EF (− 4%, *p* = 0.01, limits of agreement [− 23%, 15%]).

**Fig. 4:**
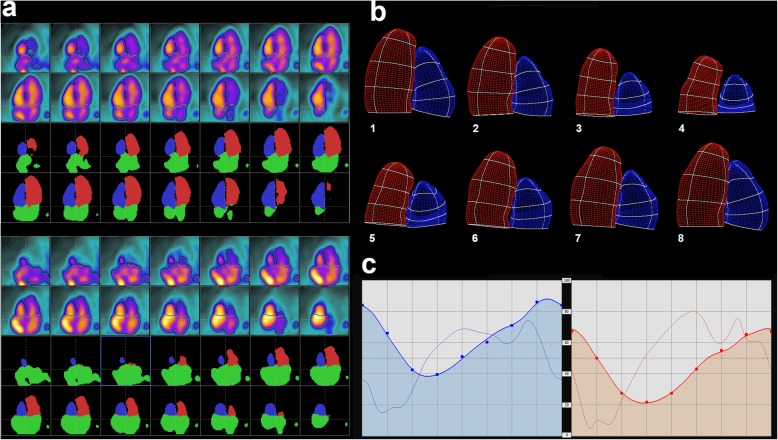
Example of a rest first-pass ^15^O-water PET study post-processed using TomPool. **a** Horizontal long-axis slices and corresponding segmentation (red: LV, blue: RV, green: extra-ventricular activity) at end-diastole (top) and end-systole (bottom). **b** Parametric surfaces built from the segmentation masks (red: LV, blue: RV) at each gating time sample. **c** Time-volume curves for LV (red) and RV (blue) computed using the deformable curve model

Figure 4 is built up of screen captures from TomPool after post-processing a rest first-pass ^15^O-water PET study. It displays the horizontal long-axis slices and corresponding segmentation (red: LV, blue: RV, green: extra-ventricular activity) at end-diastole and end-systole, the parametric surfaces built from the segmentation masks (red: LV, blue: RV) at each gating time sample, and the time-volume curves for LV (red) and RV (blue) computed using the deformable curve method.

## Discussion

To our knowledge, this is the first study to investigate the feasibility of biventricular function evaluation using first-pass gated PET in humans. The proposed methodology was implemented in the frame of myocardial perfusion PET using ^15^O-water, but it is readily transposable to any PET examination using any radiopharmaceutical. One would naturally think of the utility of a simultaneous evaluation of the cardiac function in patients referred for myocardial perfusion imaging using ^82^Rb or ^13^NH_3_ PET, or for assessment of myocardial viability or cardiac sarcoidosis using ^18^FDG PET. LV function follow-up using ^18^FDG PET would also benefit oncological patients receiving cardiotoxic chemotherapy. Accordingly, in our study, injected activities (1.5 to 3 MBq/kg) were adjusted in order to match those usually prescribed in routine ^18^FDG PET. Due to a longer half-life and shorter positron range (0.6 and 1.5 versus 2.5 mm) of the isotope, ^18^FDG and ^13^NH_3_ images should benefit from higher quality in terms of signal-to-noise ratio and spatial resolution compared to those obtained using ^15^O-water. The main constraint imposed by first-pass imaging was the need for a double reconstruction of the acquired PET data, first in a dynamic mode in order to closely circumscribe the TAC peak then in a gated mode over the time range of the first-pass of ^15^O-water. This two-step method appeared essential for image quality optimization, as attested by preliminary experiments which results are not reported here. Indeed, the blood-pool contrast was clearly diminished when gated images were reconstructed over a fixed time range (30 or 60 s) due to a significant myocardial accumulation of ^15^O-water, leading to segmentation inaccuracies and systematic volume overestimations. This issue was critical because of the freely diffusible character of the employed tracer, but it might not be as crucial in the frame of ^82^Rb and ^18^FDG PET in which myocardial uptake is expected to be slower compared to tracer first-pass [28].

In-house software was employed for image post-processing owing to its ability to be appropriately upgraded and its versatility of use. The software has been validated against CMR for equilibrium gated blood-pool SPECT using a count-based approach and a fixed threshold at 30% of the maximal intensity [8]. The count-based approach relies on the assumption of a uniform activity inside cardiac ventricles. This method is supposed to account for signal blurring at the ventricle edges due to partial volume effect and motion artefacts. The 30% threshold was initially chosen for the sake of repeatability and appeared in our study to be that ensuring the closest agreement with gated myocardial SPECT. When passing from equilibrium to first-pass imaging, the uniformity assumption was reasonably maintained for what concerns LV since tracer dilution in blood flow was sufficient downstream the pulmonary circulation [29]. The fixed 30% threshold was therefore applied with respect to the maximal intensity pixel inside the LV. As regards RV, tracer dilution was not considered effective during tracer transit through right cavities, as witnessed by the signal inhomogeneity near the RV basis at end-systole due to the proximity of a right atrial filling-related “hot spot” (see Fig. 4). To account for this phenomenon, a geometrical model was used along with an adjustable threshold that was tuned by visual inspection and so as to fit as much as possible RV and LV stroke volumes.

The recruited patients were referred for myocardial perfusion PET; hence, rest and stress acquisitions were systematically performed in order to estimate rest and stress absolute blood flow and myocardial flow reserve. Regadenoson stress is known to increase the heart rate by approximately 30% in healthy subjects [30], without significant changes in blood pressure. A transient decrease in LV EF has been reported in patients with significant ischaemia [31] which was not observed in any of our patients at the time of myocardial SPECT. Therefore, PET rest and stress acquisitions were considered as two replicates of the same examination, allowing for test-retest reliability assessment. In terms of image quality, stress acquisitions were characterized by slightly shorter transit times due to heart rate increase and lower count statistics due to both shorter transit time and lower injected activities. As appreciable from Table 3 and Figs. 1 and 2, rest and stress measurements were highly correlated and concordant. No systematic difference was observed in terms of LV and RV volumes. Minimal EF increase at stress was noted for both LV (+ 3%) and RV (+ 4%), likely due to a mild adrenergic reaction related to the discomfort following regadenoson injection, and consistent with recently reported results [32]. RV and LV stroke volumes were in close agreement, owing to the employed thresholding method for RV segmentation. Compared to LV, RV volumes were slightly higher and RV EF slightly lower, in line with previously published reference values in healthy subjects [33, 34].

In our study, myocardial perfusion SPECT was used as the reference standard for LV function assessment. This decision was based on a large body of evidence regarding the accuracy and reproducibility of this technique using both Anger [35] and CZT cameras [36, 37]. The choice of the post-processing tool relied on the fact that Corridor4DM yields minimal bias in volume measurements with respect to other commercial software and no significant difference in EF estimations with a comparison to CMR [38]. First-pass PET measurements correlated well with SPECT measurements, especially for LV volumes (Pearson’s correlation around 0.9), with similar correlation coefficients for rest and stress studies (results not shown). The low resolution of SPECT images is known to induce ESV underestimation that accounts for the observed difference with PET estimations (4 mL). As regards EF estimations, first-pass PET resulted in a minimal underestimation (− 4%) that remained within the reported range of variability of SPECT LV EF estimations [39]. Of note, most of the outliers observed on the Bland-Altman diagrams (5 out of 9, labelled using red arrows in Fig. 3) correspond to a single patient with a significant apical scar from prior infarct, a condition in which gated myocardial SPECT results are subject to caution.

Finally, it has to be noted that stress dynamic PET imaging was started within 1 min after the completion of regadenoson injection whereas stress gated SPECT was acquired after the completion of dynamic SPECT imaging required for the WATERDAY study, i.e., 6–7 min after regadenoson injection. Theoretically, this may have an impact on LV function assessment using post-stress SPECT imaging. However, Thomas et al. recently demonstrated that the small increase in LV and RV EF observed after regadenoson injection persists during at least 15 min [32]. Consequently, we believe that this 6–7 min delay would have only a negligible effect on the results of gated SPECT LV function assessment in this study.

## Conclusions

These preliminary results demonstrate that complete LV function assessment is feasible using first-pass gated PET. LV volume and ejection fraction measurements showed satisfying repeatability and are in close agreement with those provided by gated myocardial SPECT. Right ventricular function assessment is also workable, although no reference standard was available for comparison in the present study.

The feasibility of biventricular function assessment using first-pass gated ^15^O-water PET should favour the future development of similar first-pass methods using routine tracers as ^82^Rb. Such methods should benefit from the high resolution and sensitivity of last generation PET scanners and would substantially expand the diagnostic information provided by routine cardiac PET explorations.
